# Nutrition and Depression: A Narrative Review of Biological Mechanisms and Evidence from Observational Studies and Clinical Trials

**DOI:** 10.3390/nu18213479

**Published:** 2026-10-23

**Authors:** Sunhye Shin, Chan Yoon Park

**Affiliations:** 1 Department of Food and Nutrition, College of Science and Convergence Technology, Seoul Women’s University, Seoul 01797, Republic of Korea; sshin@swu.ac.kr; 2 Major in Food and Nutrition, School of Bio-Health Convergence, Sungshin Women’s University, Seoul 01133, Republic of Korea

**Keywords:** depression, nutritional psychiatry, dietary patterns, micronutrients, neuroinflammation, immunometabolism, Mediterranean diet, vitamin D

## Abstract

Depression is one of the most prevalent and disabling psychiatric disorders worldwide and has a heterogeneous pathophysiology involving interacting neurobiological, neuroimmune, and metabolic processes. Alterations in neurotransmission, neuroinflammation, oxidative stress, gut–brain communication, hypothalamic–pituitary–adrenal axis dysregulation, and immunometabolic homeostasis may provide biological links between nutrition and depression. This narrative review examines current evidence linking nutrition and depression across mechanistic, observational, and clinical levels. Regarding dietary patterns, observational evidence generally associates greater adherence to the Mediterranean diet with more favorable depression-related outcomes, while evidence for the DASH diet remains more limited. In contrast, Western dietary patterns and greater consumption of ultra-processed foods are associated with higher risks of depression-related outcomes. Individual nutrients, including omega-3 polyunsaturated fatty acids, vitamin D, vitamin C, folate, vitamin B12, vitamin B6, and essential minerals, have also been investigated in relation to depression, but findings from supplementation studies vary across nutrients, study populations, and baseline nutritional status. However, much of the evidence linking nutritional factors with depression is observational and does not establish causality. Clinical trials of nutrient supplementation and dietary interventions have reported potential benefits in some populations, but findings remain heterogeneous, and large prevention trials have also reported null results. Differences in baseline nutritional status, depression phenotype, and metabolic and inflammatory profiles may partly contribute to this heterogeneity. Future well-designed clinical trials, together with advances in multi-omics and precision approaches, may help identify individuals most likely to benefit from targeted nutritional strategies.

## 1. Introduction

Depression is a leading cause of disability worldwide and imposes a substantial burden on individuals, healthcare systems, and society. According to the Global Burden of Disease (GBD) Study 2019, depressive disorders ranked among the leading causes of disease burden worldwide [1]. This burden was further exacerbated by the COVID-19 pandemic, highlighting the continuing need for effective strategies for the prevention and management of depression [2,3].

The pathophysiology of depression has historically been investigated in relation to alterations in monoaminergic neurotransmission, including serotonin, norepinephrine, and dopamine signaling [4,5]. However, contemporary models recognize depression as a heterogeneous and multifactorial disorder that cannot be adequately explained by alterations in monoamine signaling alone. Current evidence implicates interacting disturbances in neurotransmission, neuroimmune and inflammatory signaling, oxidative stress, neuroplasticity, hypothalamic–pituitary–adrenal (HPA) axis regulation, gut–brain communication, and systemic metabolic homeostasis [6,7,8]. These biological processes are interconnected and may contribute to the substantial heterogeneity observed in depressive symptoms, disease course, and treatment response. Depressive presentations may span a broad spectrum of severity, from subthreshold symptoms to clinically diagnosed depressive disorders [9].

Despite advances in pharmacological and psychological treatments, the management of depression remains challenging. Therapeutic responses vary considerably among individuals, and a substantial proportion of patients experience treatment-resistant symptoms [10]. Even among previously untreated patients, remission is achieved in only approximately 50% following first-line antidepressant treatment or evidence-based psychotherapy, with substantially lower rates reported in real-world clinical settings [11]. Pharmacological treatments may also be accompanied by adverse effects that can affect adherence and long-term health [12]. These limitations underscore the need to investigate complementary approaches that may support established treatments for depression.

Nutrition has received increasing attention in this context because nutritional and metabolic factors may interact with several biological processes implicated in depression [13,14]. Nutrients and dietary patterns may influence neurotransmitter metabolism, inflammatory and oxidative pathways, mitochondrial function, gut microbial metabolism, HPA-axis regulation, and systemic immunometabolic homeostasis [15]. These interconnected pathways provide a biological framework linking nutrition and depression and have contributed to the emergence of nutritional psychiatry, an interdisciplinary field examining the relationships among nutrition, biological processes, and mental health [15,16]. However, biological plausibility and observational associations do not necessarily translate into clinical benefits. Although numerous studies have investigated nutritional factors in relation to depression, findings from randomized controlled trials (RCTs) remain heterogeneous, highlighting an important gap between proposed mechanisms, epidemiological associations, and clinical intervention evidence. Building on previous reviews of nutrients, foods, and dietary patterns in depression [13,14,15], an updated synthesis of the growing evidence may provide a broader understanding of the potential roles and limitations of nutritional approaches.

This narrative review first examines the major neurobiological, neuroimmune, and metabolic mechanisms implicated in depression, providing a biological context for understanding the potential roles of nutritional factors. It then explores dietary patterns and individual nutrients that may influence these pathways, summarizing findings from observational studies, systematic reviews, meta-analyses, and clinical trials. By considering both overall dietary patterns and their key nutritional components, this review aims to provide a more comprehensive perspective on the potential relationships between nutrition and depression. These findings are discussed in relation to depression-related outcomes and their potential clinical implications, while acknowledging inconsistencies and limitations in the available evidence. Finally, we consider how heterogeneity in nutritional status, depression phenotypes, and metabolic and inflammatory profiles may inform future precision nutrition approaches to depression.

## 2. Literature Search and Scope

This narrative review was informed by a literature search conducted primarily in PubMed, supplemented by Google Scholar, focusing on studies examining the relationship between nutrition and depression and the potential biological mechanisms involved. The literature search was last updated through September 2026. Search terms included combinations of depression-related terms (“depression,” “depressive symptoms,” and “major depressive disorder”) with terms related to dietary patterns (Mediterranean, DASH, and Western diets and ultra-processed foods) and individual nutrients (omega-3 fatty acids, vitamin D, folate, vitamin B6, vitamin B12, vitamin C, magnesium, zinc, iron, and polyphenols) covered in this review.

Priority was given to higher-level evidence, including umbrella reviews, systematic reviews, meta-analyses, RCTs, and large prospective cohort studies. Preclinical and mechanistic studies were selectively included to provide biological context and were considered separately from evidence of clinical efficacy. Given the narrative scope of this review, the literature selection was intended to provide a representative synthesis of current evidence rather than an exhaustive systematic review. Relevant studies were organized and selected based on their relevance to the review’s scope, with clearly unrelated records excluded during the initial assessment.

Mediterranean and DASH diets were selected as representative healthy dietary patterns widely recognized in dietary guidelines [17], with previous studies examining their associations with depression-related outcomes and potential effects on biological pathways implicated in depression [18,19,20]. Western dietary patterns and ultra-processed foods were included to represent contrasting dietary characteristics associated with poorer diet quality. Essential nutrients were selected to reflect key nutritional components of healthy dietary patterns and their potential biological relevance to depression. Polyphenols, although not classified as essential nutrients, were also included as important food-derived bioactive compounds characteristic of the Mediterranean diet.

The clinical interpretations presented in Table 1 and Table 2 reflect the authors’ qualitative synthesis of the available evidence, considering the study designs, the general direction of findings, consistency across studies, and the limitations of the reported outcomes. These interpretations were intended to provide a concise overview of the potential clinical relevance and limitations of each nutritional factor, rather than formal ratings of evidence quality or certainty.

## 3. Neurobiological, Neuroimmune, and Metabolic Mechanisms in Depression

**Figure 1:**
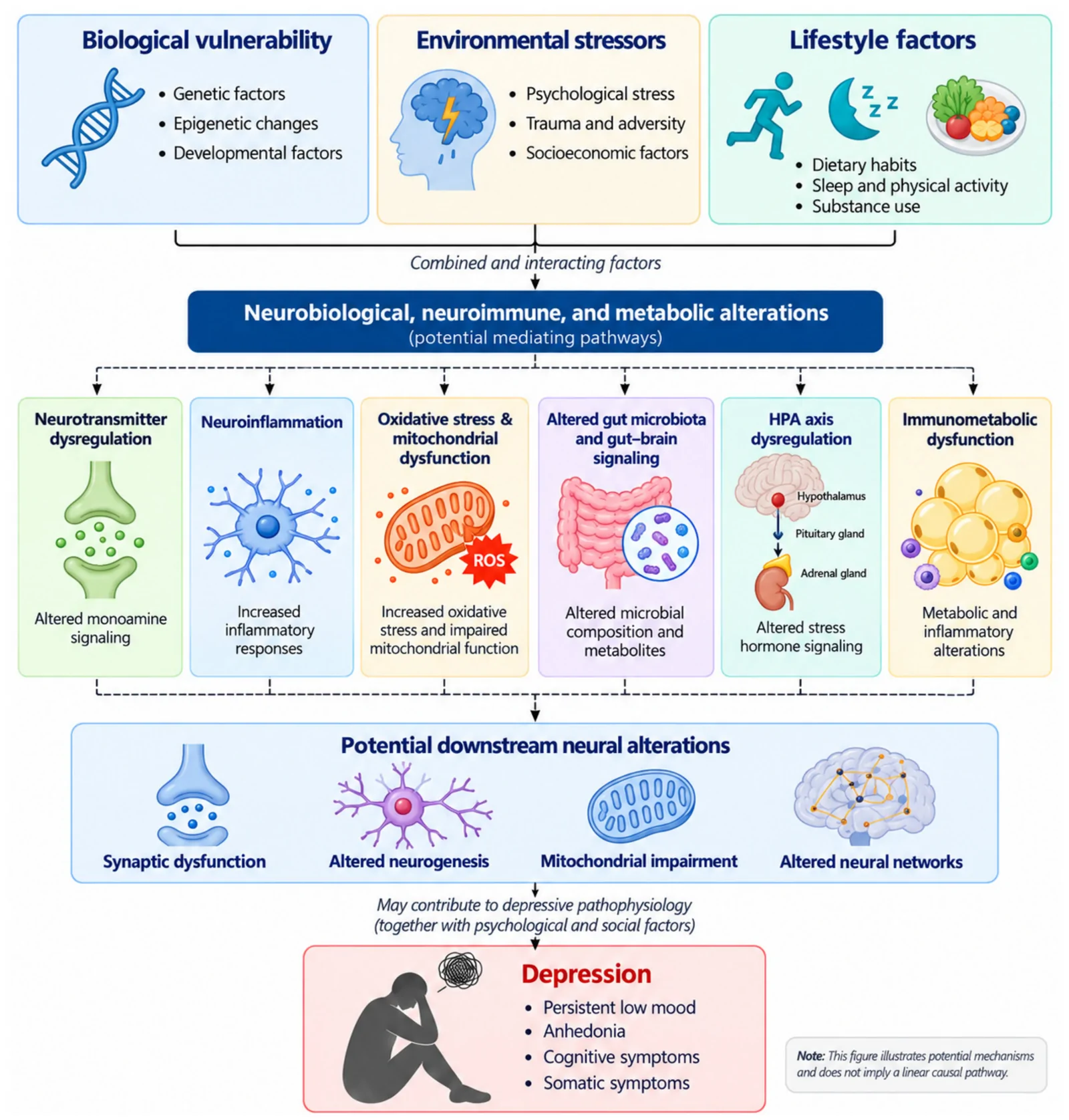
Multifactorial neuroimmune and metabolic mechanisms underlying depression (Overview).

Depression involves interacting neurobiological, neuroimmune, and metabolic processes rather than a single pathophysiological pathway that may provide biological links between nutrition and depression. Several of these processes may be influenced by nutritional and metabolic factors, providing potential biological links between nutrition and depression. The following sections summarize these mechanisms as a framework for interpreting the nutritional evidence, while recognizing that biological plausibility does not necessarily indicate clinical efficacy (Figure 1).

### 3.1. Neurotransmitter Dysregulation

Neurotransmitters are the brain’s chemical messengers for neural communication. Monoaminergic neurotransmission has long been investigated in relation to the pathophysiology of depression, with particular attention to serotonin, norepinephrine, and dopamine, which are involved in mood, motivation, reward, and emotional responses [5]. The clinical efficacy of antidepressants that modulate monoaminergic neurotransmission further stimulated research into the role of these systems in depression. However, accumulating evidence indicates that depression cannot be explained by a simple deficiency or “chemical imbalance” of monoamine neurotransmitters [6,21].

Serotonin (5-hydroxytryptamine, 5-HT) is a monoamine neurotransmitter synthesized from the essential amino acid tryptophan. Because serotonin cannot cross the blood–brain barrier (BBB), brain serotonin levels depend on the availability of circulating tryptophan [22]. Serotonin is one of the most extensively studied neurotransmitters in depression, in part because of the development and widespread clinical use of selective serotonin reuptake inhibitors (SSRIs) [5]. However, direct evidence supporting serotonergic deficiency in depression has remained inconsistent. An individual-participant meta-analysis of three cohorts involving 1869 postmenopausal women found no significant association between depression and plasma serotonin concentrations after correction for multiple comparisons, whereas antidepressant use was independently associated with lower plasma serotonin levels [23]. Similarly, a meta-analysis of cerebrospinal fluid monoamine metabolites found no significant difference in cerebrospinal fluid 5-hydroxyindoleacetic acid (5-HIAA), the principal serotonin metabolite, between individuals with depressive disorders and healthy controls [24].

Although serotonin deficiency alone cannot fully explain depression, serotonergic signaling may contribute to individual vulnerability to the disorder. A prospective cohort study by Caspi et al. initially reported that carriers of the short (S) allele of the 5-HTTLPR polymorphism were more susceptible to depression following stressful life events [25]. Similarly, a meta-analysis of monoamine depletion studies showed that acute tryptophan depletion affected only individuals vulnerable to depression, but not healthy controls, supporting the role of serotonergic dysfunction as a vulnerability factor rather than a primary cause of depression [26]. Nevertheless, an umbrella review including 17 systematic reviews, meta-analyses, and large genetic studies found no consistent associations between depression and serotonin or 5-HIAA concentrations, 5-HT1A receptor binding, serotonin transporter (SERT) binding, tryptophan depletion, or SERT gene polymorphisms, indicating that depression cannot be explained solely by serotonergic dysfunction [6].

### 3.2. Neuroinflammation

(1) Peripheral immune activation

Accumulating evidence indicates that major depressive disorder (MDD) is associated with systemic immune dysregulation characterized by chronic low-grade inflammation, altered immune cell populations, and aberrant immune activation [27]. Several meta-analyses have reported elevated peripheral levels of proinflammatory cytokines, including interleukin (IL)-6, tumor necrosis factor-α (TNF-α), and *C*-reactive protein (CRP), in patients with MDD [28,29]. A meta-analysis involving 3212 patients with MDD and 2798 healthy controls further found increased peripheral levels of IL-6, TNF-α, IL-10, soluble IL-2 receptor (sIL-2R), C-C motif ligand 2 (CCL2), IL-13, IL-18, IL-12, IL-1 receptor antagonist (IL-1RA), and soluble TNF receptor 2 (sTNFR2), whereas interferon-γ (IFN-γ) levels were reduced [29]. Consistent with these protein-level findings, peripheral blood mononuclear cells (PBMCs) from patients with MDD exhibit increased expression of inflammatory genes, including IL-1β, IL-6, TNF-α, macrophage migration inhibitory factor (MIF), and IFN-γ, whereas IL-4 expression is decreased [30].

These peripheral inflammatory alterations are accompanied by aberrant activation of innate immune signaling pathways, particularly the toll-like receptor 4 (TLR4)–nuclear factor kappa B (NF-κB) axis and the NOD-, LRR- and pyrin domain-containing protein 3 (NLRP3) inflammasome, thereby amplifying pro-inflammatory cytokine production and perpetuating chronic inflammation [31,32]. Consistent with these findings, increased expression of NLRP3 and caspase-1 has been observed in circulating immune cells from patients with MDD, accompanied by elevated circulating levels of IL-1β and IL-18 [33]. Notably, antidepressant treatment attenuates NLRP3 inflammasome activation and reduces IL-1β and IL-18 levels, although it remains unclear whether these immunological changes directly mediate clinical improvement [34].

In addition to altered cytokine profiles, MDD is associated with changes in immune cell composition and function. Patients with depression exhibit increased circulating leukocytes, neutrophils, monocytes, and altered T-cell subsets, including an increased CD4/CD8 ratio and elevated Th17 cells, suggesting alterations in both innate and adaptive immune responses [35,36].

(2) Peripheral-to-central immune communication and microglial activation

Peripheral immune activation may influence the central nervous system (CNS) inflammation through multiple neuroimmune communication pathways. Circulating cytokines can access the brain through active transport mechanisms, neural signaling pathways, or regions with a compromised BBB [37]. In addition, chronic systemic inflammation has been associated with increased BBB permeability, which may facilitate the infiltration of immune cells into the CNS [38]. Consistent with increased BBB permeability, neuroimaging and postmortem studies have reported evidence of altered central inflammatory activity in MDD, including increased translocator protein-targeted PET binding, altered IL-6 and TNF-α levels in the cerebrospinal fluid and brain parenchyma, and increased microglial activation [39].

### 3.3. Oxidative Stress

Oxidative stress is defined as an imbalance between the production of reactive oxygen species (ROS) and antioxidant defense mechanisms. Accumulating evidence suggests that oxidative stress contributes to the development and progression of depression. Excessive ROS generation and an inefficient antioxidant response can promote inflammation, lipid and protein damage, mitochondrial dysfunction, and neuronal injury [40]. Increased brain lipid peroxidation and alterations in nitric oxide (NO) and cyclooxygenase-2 (COX-2)-related pathways have also been reported in association with depression [41]. Oxidative stress may also interact with other biological processes implicated in depression, including HPA axis dysregulation, neuroinflammation, neurotransmitter imbalance, and impaired neurogenesis and synaptic plasticity [42]. These reciprocal interactions suggest that oxidative stress may form part of a broader network of biological alterations associated with depression rather than acting as an isolated causal mechanism.

### 3.4. Gut Microbiota

The gut microbiota has emerged as a key regulator of the gut–brain axis, mediating bidirectional communication between the gastrointestinal tract and the CNS [43]. Growing evidence suggests that the gut microbiota may influence biological processes relevant to depression through multiple interconnected pathways, including neurotransmitter metabolism, immune regulation, HPA axis modulation, and gut–brain signaling [44]. Experimental support for microbiota-brain communication comes partly from germ-free (GF) mouse models, which exhibit altered HPA axis activity and monoaminergic neurotransmission [45]. However, the relevance of findings from these animal models to human depression remains uncertain.

Disruption of intestinal barrier integrity is a proposed mechanism linking gut microbial alterations to depression. Increased intestinal permeability, often referred to as “leaky gut” may facilitate the translocation of bacterial products, including lipopolysaccharide (LPS) derived from Gram-negative bacteria, into the circulation, potentially promoting systemic immune activation [43,46]. Supporting this hypothesis, patients with MDD have been reported to exhibit elevated serum IgA and IgM responses to LPS derived from enterobacteria, suggesting increased bacterial translocation and immune activation [47].

Human studies of the gut microbiota in depression have yielded heterogeneous findings, and a consistent depression-specific microbial profile has not been established [48,49,50]. Nevertheless, several studies have reported alterations in short-chain fatty acid (SCFA)-producing bacteria, including *Coprococcus* and *Faecalibacterium*, as well as changes in microbial functions related to SCFA production and tryptophan metabolism [48,49,51]. These functional alterations have been proposed to influence inflammatory and neuroimmune pathways relevant to depression, although their directionality and causal relevance in humans remain uncertain [52]. Evidence from microbiota-targeted interventions, including probiotics, remains limited and heterogeneous, with uncertainty regarding their clinical efficacy and effects on gut microbiota in patients with depression [53].

### 3.5. HPA Axis

The HPA axis is the principal neuroendocrine system regulating the physiological response to stress. Activation of the HPA axis stimulates the sequential release of corticotropin-releasing hormone (CRH), adrenocorticotropic hormone (ACTH), and cortisol, which subsequently exerts negative feedback through glucocorticoid receptors (GRs) and mineralocorticoid receptors (MRs) [54,55]. In MDD, impaired GR-mediated negative feedback and altered CRH signaling have been associated with HPA axis dysregulation and altered cortisol responses to stress [55,56]. Importantly, cortisol dysregulation appears to be more closely associated with acute, severe, melancholic, and psychotic depression than with chronic or atypical depression, suggesting that HPA axis hyperactivity reflects specific depressive phenotypes rather than depression as a whole [56].

### 3.6. Immunometabolic Mechanisms

Immunometabolism refers to the reciprocal interaction between immune signaling and cellular metabolism, whereby metabolic reprogramming of immune cells influences inflammatory responses. Growing evidence suggests that immunometabolic dysfunction is implicated in depression and frequently co-occurs with obesity, insulin resistance, dyslipidemia, type 2 diabetes [57,58], and cardiovascular disease, reflecting shared biological features including chronic low-grade inflammation, metabolic dysfunction, and altered energy homeostasis [36,59,60,61].

Recent evidence suggests that these immunometabolic alterations may be particularly prominent in a subset of patients with depression, leading to the concept of immunometabolic depression (IMD) [62]. IMD has been proposed as a depressive phenotype characterized by the co-occurrence of atypical and energy-related symptoms (e.g., hypersomnia, fatigue, and hyperphagia), systemic low-grade inflammation, and metabolic dysregulation [63]. Individuals with IMD frequently exhibit obesity, dyslipidemia, insulin resistance, and leptin resistance, consistent with immune–metabolic dysregulation. Individuals with IMD are at increased risk of developing cardiometabolic diseases [61]. Immunometabolic features have also been associated with poorer response to conventional antidepressant treatment [64]. These findings are consistent with the biological heterogeneity of depression and suggest that immunometabolic characteristics may contribute to differences in disease course and treatment response.

## 4. Dietary Patterns and Depression

Modern food systems have undergone substantial changes, characterized by increased availability and consumption of energy-dense, nutrient-poor, and highly processed foods while reducing the availability and accessibility of nutrient-rich foods [65]. This dietary transition has become a major public health concern, with a poor diet contributing substantially to the global burden of non-communicable diseases [66]. Against this background, increasing attention has focused on the potential role of overall dietary patterns in mental as well as physical health.

**Figure 2:**
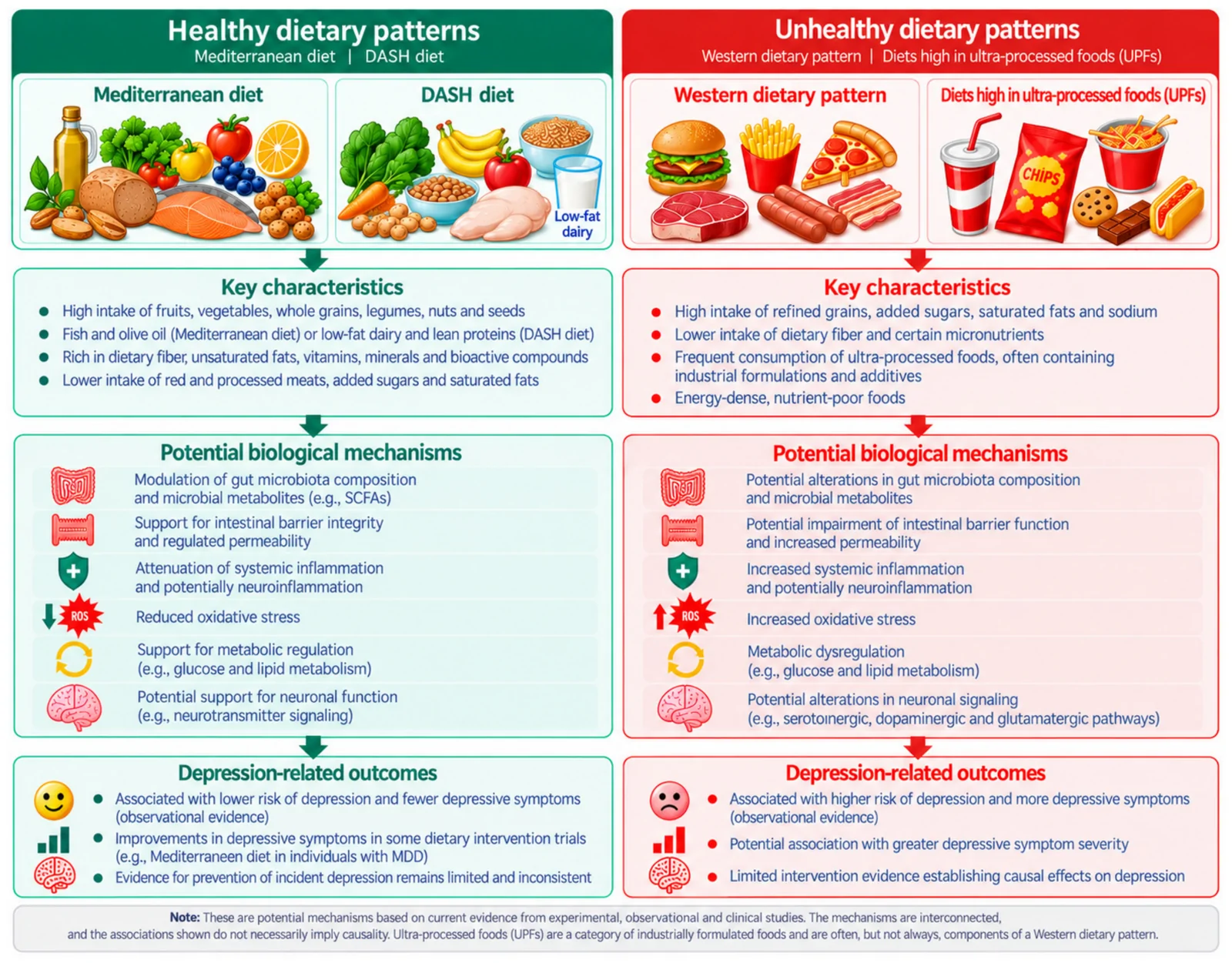
Healthy and unhealthy dietary patterns modulate neuroimmune and metabolic pathways relevant to depression.

Observational studies have examined associations between overall dietary patterns and depression, while clinical trials have investigated whether dietary modification can improve depressive symptoms or prevent the onset of depressive disorders. Importantly, these findings represent different levels and types of evidence, requiring a clear distinction between observational associations, treatment of established depression, and prevention of incident depression. Dietary patterns may also interact with biological processes implicated in depression, including inflammatory and metabolic pathways, oxidative stress, and gut–brain communication; however, the extent to which these mechanisms mediate associations or clinical effects in humans remains incompletely established (Table 1 and Figure 2).

### 4.1. Mediterranean Diet

The Mediterranean diet, one of the most representative healthy eating patterns, emphasizes whole grains, nuts, fruits, vegetables, fish, low-fat dairy products, and olive oil and limits red meat, sweets, and butter. Therefore, it is rich in dietary fiber, mono- and polyunsaturated fatty acids, and bioactive compounds, which have antioxidant and anti-inflammatory properties [67]. The antioxidant and anti-inflammatory properties of these dietary components may influence biological pathways relevant to depression including neuroinflammation, oxidative stress, and neuronal function [68,69]. However, direct evidence that these mechanisms mediate the association between Mediterranean diet adherence and depression in humans remains limited.

Observational evidence generally supports an inverse association between Mediterranean diet adherence and depression-related outcomes. A meta-analysis of observational studies (20 longitudinal and 21 cross-sectional studies) showed that the highest category of Mediterranean diet adherence had a lower incidence of depression compared to the lowest category in healthy participants (RR, 0.67; 95% CI 0.55–0.82) [18], and a systematic review (13 studies; 3058 participants) reported that higher adherence to Mediterranean diet was associated with a lower prevalence of depression in children and adolescents [70]. Another meta-analysis demonstrated that Mediterranean diet adherence was inversely associated with the risk of depression in 3 cohort studies (overall HR, 0.81; 95% CI, 0.70–0.94) and in 8 cross-sectional studies (overall OR, 0.81; 95% CI, 0.71–0.92) [19,71]. However, these findings are observational and remain susceptible to residual confounding and reverse causality.

Evidence from intervention studies is less consistent. A meta-analysis of RCTs reported that Mediterranean diet interventions also significantly improved depressive symptoms in adults with MDD or mild to moderate depressive symptoms (5 trials; 1507 participants aged 22.0–53.3 years; SMD, −0.53; 95% CI, −0.90 to −0.16) [72], whereas another meta-analysis of RCTs reported no significant effect of short-term Mediterranean diet intervention (≤4 months) on severity of depression in individuals with MDD or elevated depression levels compared to control (5 trials; 952 participants) [73]. Taken together, observational evidence generally supports an inverse association between Mediterranean diet adherence and depression-related outcomes, whereas evidence for the clinical efficacy of Mediterranean diet interventions remains inconsistent.

### 4.2. DASH Diet

The DASH (Dietary Approaches to Stop Hypertension) diet, designed to lower blood pressure and prevent cardiovascular diseases, limits sodium, saturated fat, and added sugars, while emphasizing whole grains, lean protein, low-fat dairy products, fruits, vegetables, nuts, and legumes. The DASH diet provides several nutrients and bioactive compounds involved in antioxidant defense, neuronal signaling, and metabolic regulation [74]. In addition, potassium, magnesium, and calcium play integral roles in neuronal function. Alterations in sodium and potassium balance may affect neuronal activity, while magnesium has been investigated for its potential antidepressant effects [71].

Evidence linking the DASH diet with depression remains relatively limited. In a harmonized meta-analysis of 3 cohort studies (InCHIANTI [Italy], Whitehall II [United Kingdom], and ALSWH [Australia]; 10,721 participants; 5–6 years of follow-up), DASH adherence was negatively correlated with depressive symptoms (β, −0.03; 95% CI, −0.05 to −0.01) and high DASH score was inversely associated with incidence of high depressive symptoms as a dichotomous variable (OR, 0.90; 95% CI, 0.84–0.97) [20]. The DASH intervention group had lower depression scores compared to the control group in women with migraine (102 participants; aged 20–50 years; 12-week intervention; DASH vs. control [mean ± standard error of mean], 10.39 ± 1.10 vs. 15.73 ± 1.43; *p* = 0.011) [75] and in postmenopausal women with type 2 diabetes (66 participants; 12-week intervention; DASH vs. control [mean ± standard deviation], 3.19 ± 3.44 vs. 5.97 ± 3.44; *p* < 0.0001) [76]. However, the intervention studies were conducted in specific clinical populations and were not primarily designed to evaluate the treatment of depressive disorders. Therefore, evidence supporting the DASH diet as an intervention for depression remains insufficient.

### 4.3. Western Diet

The Western dietary pattern is generally characterized by high intakes of red and processed meats, refined grains, saturated fats, and added sugars, together with relatively low intakes of fruits, vegetables, whole grains, and dietary fiber. These dietary characteristics have been associated with chronic low-grade inflammation and metabolic dysfunction, providing potential biological links with depression [77].

A systematic review and meta-analysis of prospective observational studies in adults aged ≥45 years found that greater adherence to Western dietary patterns was associated with higher odds of incident depression (OR, 1.15; 95% CI, 1.04–1.26) [78]. More recently, an updated meta-analysis of 54 observational studies reported associations of Western dietary patterns with both depression (OR, 1.19; 95% CI, 1.06–1.32) and depressive symptoms (OR, 1.20; 95% CI, 1.08–1.34) [79]. However, the evidence remains observational, and residual confounding and reverse causality cannot be excluded. Residual confounding and reverse causality remain important considerations.

### 4.4. Ultra-Processed Foods

Ultra-processed foods (UPFs), as defined by the NOVA classification, are industrial formulations that are often energy-dense and high in added sugars, saturated fats, and sodium, while being relatively low in dietary fiber and essential micronutrients [80]. Several mechanisms have been proposed to link high UPF consumption with adverse health outcomes, including poor overall diet quality, metabolic and inflammatory alterations, and potential effects of food additives and industrial processing on the gut microbiota and intestinal barrier [81]. However, the contribution of these mechanisms to depression in humans remains uncertain.

Epidemiological evidence generally supports an association between higher UPF consumption and depression-related outcomes. An umbrella review of epidemiological meta-analyses reported that higher UPF exposure was associated with a higher risk of incident depressive outcomes (HR, 1.22; 95% CI, 1.16–1.28) [82]. Prospective cohort studies further support this association while distinguishing between depressive symptoms and clinically defined depression [83,84]. In the French NutriNet-Santé cohort, each 10% increase in the proportion of UPFs in the diet was associated with a 21% higher risk of incident depressive symptoms (HR, 1.21; 95% CI, 1.15–1.27) [84]. Similarly, the Nurses’ Health Study II found that higher UPF consumption was associated with a greater risk of incident depression, defined using clinician-diagnosed depression and antidepressant use, with the association remaining in analyses addressing potential reverse causation [83]. Nevertheless, direct intervention evidence demonstrating that reducing UPF consumption prevents or improves depression remains limited, and residual confounding and reverse causality cannot be fully excluded.

### 4.5. Clinical Implications of Dietary Interventions

Clinical trials have examined whether structured dietary interventions can improve depressive symptoms or prevent the onset of depressive disorders. These represent distinct clinical outcomes and should not be interpreted interchangeably.

The SMILES trial evaluated a dietitian-supported modified Mediterranean diet (ModiMedDiet) in adults with MDD and reported greater improvements in depressive symptoms and higher remission rates than a social-support control [85]. Similarly, the HELFIMED trial reported improvements in depressive symptoms following a Mediterranean-style dietary intervention supplemented with fish oil [86]. However, both trials were relatively small, and their findings primarily concern improvement in depressive symptoms among individuals with existing depression rather than prevention of incident MDD.

In contrast, in the large MoodFOOD RCT involving 1025 overweight or obese adults with subsyndromal depressive symptoms, neither multinutrient supplementation nor food-related behavioral activation significantly reduced the onset of MDD over 12 months [87]. These findings highlight the importance of distinguishing treatment of established depression, adjunctive management of depressive symptoms, and prevention of incident MDD when interpreting nutritional intervention studies.

Overall, dietary interventions have shown potential benefits for depressive symptoms in some populations, although findings remain heterogeneous and evidence for their clinical efficacy is not yet conclusive. Further well-designed trials are needed to clarify their potential role in the adjunctive management and prevention of depression.

**Table 1:** Selected evidence on dietary patterns and depression-related outcomes.

Dietary Patterns	Study Designs	Sample/Populations	Exposure/Intervention	Outcomes	Clinical Interpretation ^1^	References
Mediterranean diet (MD)	Meta-analysis	20 longitudinal and 21 cross-sectional studies	MD adherence	The highest category of MD adherence had the lower incidence of depression compared to the lowest (RR, 0.67; 95% CI, 0.55–0.82).	Consistent observational associations; conflicting intervention evidence	[18]
	Systematic review (SR)	13 studies; n = 3058; children and adolescents	MD adherence	Higher MD adherence was associated with the lower prevalence of depression in children and adolescents.		[70]
	Meta-analysis	3 cohort studies and 8 cross-sectional studies	MD adherence	MD adherence was inversely associated with depression risk both in cohort (HR, 0.81; 95% CI, 0.70–0.94) and in cross-sectional studies (OR, 0.81; 95% CI, 0.71–0.92).		[19,71]
	Meta-analysis	5 RCTs; n = 1507; adults with MDD or mild to moderate depressive symptoms	MD intervention	MD improved depressive symptoms in adults with MDD or mild to moderate depressive symptoms (SMD, −0.53; 95% CI, −0.90 to −0.16).		[72]
	Meta-analysis	5 RCTs; 952 participants with MDD or elevated depression levels	MD intervention	Short-term MD intervention (≤4 months) did not improve severity of depression in individuals with MDD or elevated depression levels.		[73]
DASH diet	Meta-analysis	3 cohort studies; n = 10,721; 5–6 years of follow-up	DASH adherence	DASH adherence was negatively correlated with depressive symptoms (β, −0.03; 95% CI, −0.05 to −0.01). High DASH score was inversely associated with incidence of elevated depressive symptoms (OR, 0.90; 95% CI, 0.84–0.97).	Limited observational evidence; insufficient intervention evidence.	[20]
	RCT	n = 102; women with migraine; aged 20–50 years	DASH intervention; 12 weeks	DASH intervention group had lower depression scores (DASH vs. control [mean ± SEM], 10.39 ± 1.10 vs. 15.73 ± 1.43; *p* = 0.011).		[75]
	RCT	n = 66; postmenopausal women with type 2 diabetes	DASH intervention; 12 weeks	DASH intervention group had lower depression scores (DASH vs. control [mean ± SD], 3.19 ± 3.44 vs. 5.97 ± 3.44; *p* < 0.0001).		[76]
Western diet (WD)	SR and meta-analysis of cohort studies	33 studies; 21 included in meta-analyses; community-dwelling adults ≥45 y	WD pattern	Greater adherence to a WD pattern was associated with higher odds of incident depression (OR, 1.15; 95% CI, 1.04 to 1.26).	Consistent observational associations; insufficient evidence for causality	[78]
	SR and meta-analysis	54 observational studies; Mixed population	WD patterns and typical food groups	WD patterns were associated with a higher risk of depression (OR, 1.19; 95% CI, 1.06 to 1.32) and depressive symptoms (OR, 1.20; 95% CI, 1.08 to 1.34).		[79]
	Prospective cohort	n = 12,059; 657 incident cases; median follow-up 6.1 y; Spanish university graduates free of depression at baseline	Trans fatty acid intake	Higher trans fatty acid intake was associated with a greater risk of incident depression in a dose–response manner (highest vs. lowest quintile: HR, 1.42; 95% CI, 1.09 to 1.84; *p*-trend = 0.003).		[88]
Ultra-Processed Food (UPF)	Prospective cohort	n = 26,730; 2221 incident cases; mean follow-up 5.4 y; Adults aged 18–86 y without depressive symptoms at baseline	NOVA-defined UPF intake; repeated 24-h dietary records	Higher UPF consumption was associated with a greater risk of incident depressive symptoms; each 10% increase in the proportion of UPF in the diet was associated with a 21% higher risk (HR, 1.21; 95% CI, 1.15–1.27).	Consistent observational associations; insufficient intervention evidence	[84]
	SR & meta-analysis	17 studies; n = 385,541; Mixed populations	NOVA-defined UPF consumption	Higher UPF consumption was associated with greater odds of depressive symptoms in cross-sectional studies (OR, 1.44; 95% CI, 1.14–1.82) and a higher risk of depression-related outcomes in prospective studies (HR, 1.22; 95% CI, 1.16–1.28).		[89]
	Prospective cohort	n = 31,172; Middle-aged women free of depression at baseline; Nurses’ Health Study II	NOVA-defined UPF intake; repeated FFQs	Higher UPF consumption was associated with a greater risk of incident depression (highest vs. lowest quintile: HR, 1.49; 95% CI, 1.26 to 1.76); the association persisted in a 4-year lag analysis (HR, 1.32; 95% CI, 1.13 to 1.54).		[83]
	Umbrella review of epidemiological meta-analyses	45 pooled analyses; 9,888,373 participants across multiple health outcomes	NOVA-defined UPF consumption	Higher UPF consumption was associated with a greater risk of incident depressive outcomes (HR, 1.22; 95% CI, 1.16 to 1.28); evidence was classified as Class II (highly suggestive), with low GRADE certainty.		[82]
	Target trial emulation	n = 11,192; Community-dwelling adults ≥70 y in Australia	NOVA-defined UPF intake; ≥4 vs. <4 servings/day	Higher UPF consumption was associated with a greater risk of subsequent depressive symptoms (CES-D-10 ≥8; RR, 1.10; 95% CI, 1.04 to 1.18); the association remained after excluding baseline antidepressant users (RR, 1.11; 95% CI, 1.04 to 1.20).		[90]

^1^ Clinical interpretation represents qualitative narrative assessments based on the type, consistency, and limitations of the available evidence and are not formal certainty-of-evidence ratings. DASH, Dietary Approaches to Stop Hypertension; FFQ, food frequency questionnaire; HR, hazard ratio; MD, Mediterranean diet; MDD, major depressive disorder; OR, odds ratio; RCT, randomized controlled trial; RR, risk ratio; SD, standard deviation; SEM, standard error of the mean; SR, systematic review; UPF, ultra-processed food; WD, Western diet.

## 5. Individual Nutrients and Depression

Nutrients are involved in several biological processes relevant to depression, including neurotransmitter metabolism, neuroinflammation and oxidative stress, mitochondrial function, and neurogenesis [14,91]. However, biological plausibility and observational associations do not necessarily indicate the clinical efficacy of supplementation. Evidence from intervention studies varies across nutrients and study populations (Table 2).

### 5.1. Omega-3 Fatty Acids

Omega-3 (*n*-3) fatty acids, including α-linolenic acid (ALA), docosahexaenoic acid (DHA), and eicosapentaenoic acid (EPA), modulate neurogenesis, neuroinflammation, and neurotransmission; therefore, brain function is dependent on adequate levels of *n*-3 fatty acids. As a key component of brain cell membranes, *n*-3 fatty acids are critical for the integrity and fluidity of neuronal membranes. They can stimulate neuronal cell membrane expansion and neurite outgrowth, and enhance neurotransmitter binding to the receptors [92]. Also, as a precursor of anti-inflammatory eicosanoids, *n*-3 fatty acids improve metabolic processes and immune functions of the central nervous system [15]. They downregulate pro-inflammatory cell signaling, such as NF-κB, and activate neurologically beneficial signaling systems, including G protein-coupled receptor (GPR) 40, GPR120, and peroxisome proliferator-activated receptors (PPARs) [93].

Multiple studies demonstrated the therapeutic potential of *n*-3 fatty acids. A network meta-analysis (192 trials; 17,437 participants; 44 nutraceuticals) estimating the comparative efficacy demonstrated that EPA and DHA exerted better efficacy either as monotherapies (standardized mean difference [SMD], 0.60; 95% confidence interval [CI], 0.32–0.88) or as adjuncts to antidepressants (SMD, 1.04; 95% CI, 0.64–1.44) compared to antidepressants alone [94]. A prospective cohort study with Japanese older adults (aged 63–82 years; 1181 participants) reported that the 3rd quartile for fish intake (odds ratio [OR], 0.44; 95% CI, 0.23–0.84) and the 2nd quartile for EPA (OR, 0.54; 95% CI, 0.30–0.99) had a lower risk of MDD compared to their own 1st quartile [95].

However, the beneficial effects of *n*-3 fatty acids on depression are still conflicting. In an umbrella review including 22 meta-analyses with 26 effect sizes (ES), 16 ES showed improving effects of *n*-3 fatty acids on depression symptoms whereas others did not [96]. Although a meta-analysis of RCTs showed that supplementation of *n*-3 fatty acids reduced severity of depression in adults (53 trials; 10,273 participants; SMD, −1.38; 95% CI, −1.69 to −1.07) but did not reduce the risk of depression in the general population (14 trials; 32,755 participants; OR, 0.95; 95% CI, 0.79–1.15) [97]. Another meta-analysis of prospective cohort studies (10 cohort studies; 6672 cases among 109,764 participants) showed that the highest category of fish consumption had a lower risk of depression (relative risk [RR], 0.89; 95% CI, 0.80–0.99) but that of *n*-3 intake did not (RR, 0.89; 95% CI, 0.75–1.04) compared to their lowest category [98]. In an RCT with mentally healthy older adults (aged ≥50 years; 18,353 participants without depression or depressive symptoms at baseline), supplementation of marine *n*-3 fatty acids over 5 years (1 g/day fish oil containing 465 mg EPA and 375 mg DHA) did not improve longitudinal mood scores and increased depression risk (HR, 1.13; 95% CI, 1.01–1.26) compared to placebo [99]. These inconsistent results do not support the usage of *n*-3 fatty acid supplements for preventing depression.

### 5.2. Vitamin D

Vitamin D, after sequential hydroxylation to its active form 1,25-dihydroxyvitamin D [1,25(OH)2D], exerts diverse biological functions beyond calcium and bone homeostasis, including the regulation of immune and nervous system function [100]. Vitamin D receptors (VDRs) are widely distributed across brain regions involved in mood regulation and are expressed in both neuronal and non-neuronal cells [101,102,103]. In addition, both 25(OH)D and 1,25(OH)2D can cross the BBB and interact with VDRs within the central nervous system [104]. Through genomic and non-genomic mechanisms, vitamin D may influence neuroinflammation, neurotransmitter function, neurotrophic signaling, oxidative stress, and neuronal plasticity, providing biological plausibility for its potential role in depression [100,105].

Vitamin D was shown to attenuate neuroinflammation by suppressing pro-inflammatory cytokines, including TNF-α, IL-6, and IL-1β, while enhancing neuroprotective factors such as brain-derived neurotrophic factor (BDNF), vascular endothelial growth factor (VEGF), and insulin-like growth factor-1 (IGF-1), thereby promoting neuronal survival and synaptic plasticity [106]. In the brain, vitamin D also modulates microglial activation by enhancing IL-10 expression and suppressing TNF-α production [107,108]. However, the extent to which these mechanistic effects translate into improvements in depressive outcomes in humans remains uncertain.

Consistent with these mechanistic findings, observational evidence generally supports an inverse association between serum 25(OH)D concentrations and depression-related outcomes, although much of the available evidence is derived from cross-sectional studies, limiting causal inference [109,110,111]. Prospective cohort studies further support this association [109,112]. A dose–response meta-analysis of six prospective cohort studies involving 16,287 older adults reported that each 10-ng/mL increase in serum 25(OH)D concentration was associated with a 12% lower risk of incident depression (hazard ratio [HR] = 0.88; 95% CI, 0.78–0.99), although substantial between-study heterogeneity was present [112]. A recent Mendelian randomization (MR) study found no strong evidence for a causal effect of 25(OH)D on MDD using conventional MR methods, although additional analyses suggested a possible protective association with recurrent MDD [113].

Meta-analytic evidence generally supports a beneficial effect of vitamin D supplementation on depressive symptoms, although the magnitude of the effect varies across studies [114,115]. An umbrella meta-analysis combining 10 previous meta-analyses of RCTs reported an overall reduction in depressive symptoms with vitamin D supplementation (pooled SMD, −0.40; 95% CI, −0.60 to −0.21) [114]. Similarly, a meta-analysis of 41 placebo-controlled RCTs involving 53,235 adults found a modest improvement in depressive symptoms, although heterogeneity was high [115]. A meta-analysis of 18 RCTs reported a small overall improvement in depressive symptoms following vitamin D supplementation (SMD, −0.15; 95% CI, −0.26 to −0.04) [116]. However, a subsequent critical appraisal and reanalysis identified methodological concerns and found no statistically significant benefit in the reanalyzed subgroups, questioning the robustness of the original findings [117]. The original meta-analysis also included the large VITAL-DEP trial, which reported no preventive benefit of long-term vitamin D3 supplementation [118]. More recently, a 5-year RCT involving 2434 largely vitamin D-sufficient older adults found no significant reduction in incident MDD with vitamin D3 supplementation [119]. Individual RCTs in clinical depression have also reported null findings [120,121]. Taken together, intervention evidence remains heterogeneous, with possible modest benefits for depressive symptoms in some populations but no clear evidence that vitamin D supplementation prevents depression-related outcomes.

### 5.3. Folate, Vitamin B12, and Vitamin B6

Folate, vitamin B12, and vitamin B6 are involved in biological processes relevant to neurological and psychological function by regulating one-carbon metabolism. One-carbon metabolism, a network that supplies methyl groups, is involved in DNA and histone modifications, myelin maintenance, and neurotransmitter synthesis and degradation [122]. In the network, folate and vitamin B12 recycle homocysteine and generate S-adenosylmethionine (SAM), which is the primary methyl donor in the body, and vitamin B6 promotes the conversion of homocysteine to cysteine. Inadequate status of these vitamins may reduce SAM availability, which impairs methylation capacity, and increase the concentration of homocysteine, which leads to oxidative stress, neuronal dysfunction, and psychological disorders [123]. In particular, folate or vitamin B12 deficiency is shown to impair the adrenergic, glutamatergic, serotonergic, and dopaminergic systems, which may lead to depression [124,125,126], and vitamin B6, as a cofactor of amino acid metabolism, is also involved in the synthesis of neurotransmitters, including γ-aminobutyric acid (GABA), serotonin, and norepinephrine [127,128].

A meta-analysis of 13 observational studies reported that individuals in the highest categories of dietary vitamin B6 (risk ratio [RR], 0.81; 95% CI, 0.71–0.93) and vitamin B12 intake (RR, 0.86; 95% CI, 0.75–0.99) had a lower risk of depression than those in the corresponding lowest categories [129]. Another meta-analysis of 43 observational studies (8519 participants with depression and 27,282 participants without depression) reported lower folate levels in individuals with depression than in those without depression (Hedges’ g, −0.24; 95% CI, −0.31 to −0.16) [130].

Folic acid or L-methylfolate supplementation was shown to decrease depression score scales in a meta-analysis with 20 RCTs (2256 participants aged ≥18 years with MDD or mild to severe depressive symptoms) [131]. A meta-analysis of RCTs reported that adjunctive folic acid or L-methylfolate supplementation reduced depressive symptom scores, both in the analysis of Hamilton Depression Rating Scale (HDRS) scores (MD, −2.16; 95% CI, −3.62 to −0.69) and in the pooled analysis combining HDRS and Beck Depression Inventory-II outcomes (SMD, −0.61; 95% CI, −0.97 to −0.24) [132].

Although prolonged consumption (several weeks to years) of folate and vitamin B12 decreased the onset of clinical symptoms in people at risk of depression (RR, 0.65; 95% CI, 0.43–0.98) or the risk of relapse (OR, 0.33; 95% CI, 0.12–0.94), short-term use (days to few weeks) of the vitamins did not improve depressive symptoms in adults with MDD treated with antidepressants in a meta-analysis of 11 RCTs [133]. Also, in a meta-analysis of 16 RCTs with 6276 participants, supplementation of folate and vitamin B12 with or without vitamin B6 was shown to exert no effect on measures of depression [134]. Therefore, to utilize folate, vitamin B12, and vitamin B6 supplements for preventing depression, more evidence is required.

### 5.4. Vitamin C

Vitamin C regulates the function of the central nervous system, including neuron differentiation, myelin formation, catecholamine synthesis, and antioxidative protection [135]. As a key antioxidant in the brain, vitamin C exerts neuroprotective effects by scavenging reactive oxygen and nitrogen species produced by metabolism of mitochondrial neurons and by recycling other antioxidants, including α-tocopherol [136]. As a cofactor, vitamin C participates in the synthesis of neurotransmitters, in particular dopamine and norepinephrine [137].

A systematic review (9 studies; mean number of participants, 280) reported the link between vitamin C deficiency and depression [138], and a meta-analysis of observational studies (25 studies; 91,666 participants) demonstrated that dietary vitamin C intake was lower in participants with depression compared to control subjects (weighted mean difference [WMD], −11.58; 95% CI, −14.88 to −8.29) and inversely associated with the risk of depression (RR, 0.72; 95% CI, 0.57–0.91) [139]. A very recent meta-analysis showed that patients with depression had a lower vitamin C intake (SMD, −0.18; 95% CI, −0.24 to −0.13) and lower blood vitamin C level (SMD, −0.52; 95% CI, −0.94 to −0.10) compared to controls [140]. However, vitamin C supplementation was not effective in improving mood status in a meta-analysis of RCTs (10 trials; 836 participants) while it exerted beneficial effects in participants with subclinical depressive symptoms but not prescribed antidepressants (sub-group analysis of 5 trials; Hedges’ g, −0.18; 95% CI, −0.35 to −0.01) [141]. Since the evidence is inconsistent and insufficient, additional studies are required to consider the utilization of vitamin C supplements for depression.

### 5.5. Magnesium

Magnesium, which acts as a cofactor for numerous enzymes, is required for the normal functions of the central nervous system and contributes to the regulation of neuronal excitability [142]. Magnesium has also been implicated in the pathophysiology of depression through its involvement in glutamatergic neurotransmission, particularly as an endogenous antagonist of the *N*-methyl-D-aspartate (NMDA) receptor, as well as in neuroplasticity, oxidative stress, and inflammatory responses [143]. Experimental studies further support this relationship, as mice fed a magnesium-deficient diet had depression and behavioral deficits [144].

Epidemiological evidence generally supports an inverse association between magnesium intake and depression, although findings vary according to study design. A recent GRADE-assessed systematic review and dose–response meta-analysis including 63,214 adults found that individuals with the highest dietary magnesium intake had a 34% lower risk of depression than those with the lowest intake (RR, 0.66; 95% CI, 0.57–0.78), with each 100 mg/day increase in magnesium intake associated with a 7% lower risk (RR, 0.93; 95% CI, 0.90–0.96) [145]. However, an earlier meta-analysis found this inverse association in cross-sectional but not prospective studies [146].

Intervention studies also suggest a potential benefit of magnesium supplementation, although findings remain inconsistent. A meta-analysis of seven RCTs involving 325 adults with depressive disorders reported a significant reduction in depression scores (SMD, −0.92; 95% CI, −1.44 to −0.40), with a beneficial effect also reported in a placebo-controlled trial of patients with magnesium deficiency [147]. However, earlier meta-analytic and placebo-controlled evidence reported no significant benefit [146,148]; thus, the antidepressant efficacy of magnesium remains uncertain.

### 5.6. Zinc

Zinc is essential for biochemical processes related to neuroplasticity and neurotransmission in the brain. Zinc transporters and zinc-sensing receptors play significant roles in neuronal proliferation and maturation, serotonin synthesis and signaling, and responses to antidepressant treatment [149]. The antioxidant and anti-inflammatory properties of zinc may contribute to its neuroprotective effects, while zinc deficiency can alter glutamatergic and serotonergic neurotransmission and BDNF signaling [150,151].

Observational evidence generally supports an inverse association between zinc status and depression. A meta-analysis of nine studies found that higher dietary zinc intake was associated with a lower risk of depression (RR, 0.67; 95% CI, 0.58–0.76) [150]. More recently, a systematic review and meta-analysis reported significantly lower serum zinc levels in individuals with MDD than in healthy controls (SMD, −0.62; 95% CI, −0.78 to −0.46) [152]. Intervention studies also suggest a potential benefit of zinc supplementation, particularly as an adjunct to antidepressant treatment. A systematic review of controlled trials reported beneficial effects of zinc supplementation on depressive symptoms, including when used as an adjunct to conventional antidepressant therapy [153]. Consistent with this evidence, current WFSBP/CANMAT guidelines provisionally recommend adjunctive zinc (++) for unipolar depression [154]. However, the intervention evidence is based on a relatively limited number of trials, and the independent antidepressant efficacy of zinc supplementation remains uncertain.

### 5.7. Iron

Iron, a cofactor for various proteins and enzymes, plays an essential role in myelin maintenance, neurotransmitter synthesis, and oxygen transport [155]. Alterations in iron homeostasis may affect brain function through impaired neuronal energy metabolism, neurotransmission, and oxidative balance [156]. Iron deficiency in early life alters the regulation of genes related to neuronal energy metabolism and dendritic maturation [157], and iron overload induces neuronal oxidative stress and cell death [158].

Observational evidence suggests an association between lower iron intake or status and depression. A meta-analysis of three studies found that higher dietary iron intake was associated with a lower risk of depression (RR, 0.57; 95% CI, 0.34–0.95) [150]. More recently, a systematic review and meta-analysis reported significantly lower serum iron levels in individuals with MDD than in healthy controls (SMD, −0.36; 95% CI, −0.52 to −0.20) [152]. Evidence for the effects of iron supplementation on depressive symptoms is more limited and may depend on baseline iron status. Iron treatment improved depression and stress scores in postpartum women with iron-deficiency anemia [159], whereas supplementation in non-anemic women with low ferritin improved fatigue but not depression [160]. These findings suggest that potential benefits of iron supplementation may depend on baseline iron status, while evidence supporting an antidepressant effect in individuals without iron deficiency remains insufficient.

### 5.8. Polyphenols and Other Bioactive Compounds

Polyphenols are a diverse group of plant-derived bioactive compounds that have been investigated for their potential effects on neurobiological processes relevant to depression. Several polyphenols, including curcumin, resveratrol, and epigallocatechin-3-gallate, have been shown to prevent oxidative stress-induced alteration of neuron function and neurotransmission by stabilizing reactive oxygen species [161] and by reducing secretion of pro-inflammatory cytokines [162]. They were also reported to increase the level of BDNFs, essential for differentiation and survival of neurons, in the brain [163] and maintain the balance of dopamine and norepinephrine by regulating the amine pool [164].

Clinical studies have reported potential benefits of polyphenol supplementation for depressive symptoms. A meta-analysis of RCTs (18 trials; 1523 participants) reported that polyphenol supplementation improved depression scores (SMD, −0.95; 95% CI, −1.76 to −0.13) [165], and a systematic review showed that polyphenols in a Mediterranean diet were also effective (29 studies among 17 experimental and 20 observational studies) [166]. In addition, a meta-analysis of 10 studies has reported improvements in depressive symptoms following flavonoid interventions (Hedges’ g, −0.487; 95% CI, −0.814 to −0.160), with subgroup analyses suggesting greater effects at doses of 50–100 mg/day and treatment duration of ≥8 weeks [167].

Potential benefits have also been reported for specific polyphenols. A meta-analysis of six RCTs involving 377 patients with depression found that curcumin supplementation reduced HDRS scores (SMD, −0.344; 95% CI, −0.558 to −0.129) [168]. Resveratrol at 10–80 mg/kg body weight/day ameliorated depressive symptoms in rodents by decreasing neuroinflammation and by increasing BDNF and neurogenesis (a systematic review of 22 animal studies) [169]. However, clinical evidence remains heterogeneous, and the antidepressant efficacy of individual polyphenols has not been firmly established.

**Table 2:** Selected evidence on individual nutrients and depression-related outcomes.

Nutrients	Study Designs	Sample/Populations	Exposure/Intervention	Outcomes	Clinical Interpretation ^1^	References
Omega-3 fatty acids	Network meta-analysis	192 RCTs; n = 17,437	EPA and DHA supplementation	EPA and DHA had better efficacy as monotherapies (SMD, 0.60; 95% CI, 0.32 to 0.88) and as adjuncts to antidepressants (SMD, 1.04; 95% CI, 0.64 to 1.44).	Limited observational evidence; conflicting intervention evidence	[94]
	Umbrella review	22 meta-analyses with 26 effect sizes	omega-3 fatty acids	Omega-3 fatty acids improved depression symptoms in 16 effect sizes.		[96]
	Meta-analysis	67 RCTs; n = 43,028	Omega-3 fatty acid supplementation	Supplementation with omega-3 fatty acids reduced depression severity (SMD, −1.38; 95% CI, −1.69 to −1.07), but not the risk of depression in the general population (OR, 0.95; 95% CI, 0.79 to 1.15).		[97]
	Meta-analysis	10 prospective cohort studies; n = 109,764	Fish or omega-3 fatty acid consumption	The highest category of fish consumption had the lower risk of depression (RR, 0.89; 95% CI, 0.80 to 0.99), but that of *n*-3 intake did not (RR, 0.89; 95% CI, 0.75 to 1.04)		[98]
	RCT	n = 18,353; healthy older adults (aged ≥50 years)	Fish oil (1 g/day)	Supplementation with omega-3 fatty acids over 5 years did not improve longitudinal mood scores and increased the risk of depression (HR, 1.13; 95% CI, 1.01 to 1.26) compared with placebo.		[99]
	Cohort study	n = 1181; Japanese older adults (aged 63–82 years)	Dietary fish or EPA intake	The 3rd quartile for fish intake (OR, 0.44; 95% CI, 0.23 to 0.84) and the 2nd quartile of EPA (OR, 0.54; 95% CI, 0.30 to 0.99) had a lower risk of MDD.		[95]
Vitamin D	SR and meta-analysis	14 studies; n = 31,424 (1 case–control, 10 cross-sectional, 3 cohort); Adults	Serum 25(OH)D	Lower vitamin D levels were associated with depression (SMD, 0.60), and individuals in the lowest vitamin D had a higher risk of depression than those in the highest in prospective cohort studies (HR, 2.21; 95% CI, 1.40 to 3.49).	Relatively consistent observational associations; conflicting intervention evidence	[109]
	Meta-analysis of prospective cohort studies	6 prospective cohorts; n = 16,287; 1157 cases, older adults	Serum 25(OH)D	Each 10 ng/mL increase in 25(OH)D associated with 12% lower risk of incident depression (HR, 0.88; 95% CI, 0.78 to 0.99; *I*^2^, 79%).		[112]
	Umbrella meta-analysis	10 RCT meta-analyses and 4 cohort meta-analyses; mixed population	Vitamin D supplementation; serum 25(OH)D	Vitamin D supplementation reduced depressive symptoms (SMD, −0.40; 95% CI, −0.60 to −0.21) while lower serum vitamin D levels were associated with a higher risk of depression (OR, 1.60; 95% CI, 1.08 to 2.36).		[114]
	RCT meta-analysis	41 RCTs; n = 53,235; adults	Vitamin D vs. placebo	Vitamin D supplementation modestly reduced depressive symptoms compared with placebo (Hedges’ *g*, −0.317; 95% CI, −0.405 to −0.230).		[115]
	RCT meta-analysis	18 RCTs; Adults with primary depression	Vitamin D vs. placebo	Vitamin D supplementation was associated with a small reduction in depressive symptoms (SMD, −0.15; 95% CI, −0.26 to −0.04); however, a subsequent re-analysis questioned the robustness of this finding [117].		[116]
	RCT meta-analysis	31 RCTs; n = 24,189; adults	Vitamin D supplementation	Vitamin D supplementation reduced depressive symptoms (SMD, −0.32 per 1000 IU/day; 95% CI, −0.43 to −0.22) with greater effects in participants with depressive symptoms and in interventions lasting ≤ 24 weeks.		[170]
	Clinical guideline	N/A—clinical guideline, Unipolar depression	Vitamin D supplementation	Vitamin D supplementation was weakly recommended for the treatment of unipolar depression.		[154]
Folate, vitamin B12, and vitamin B6	Meta-analysis	13 observational studies	Dietary vitamin B6 and B12 intake	The highest categories of dietary vitamin B6 (RR, 0.81; 95% CI, 0.71 to 0.93) and B12 intake (RR, 0.86; 95% CI, 0.75 to 0.99) were associated with a lower risk of depression compared with their lowest categories.	Observational associations reported; conflicting intervention evidence	[129]
	Meta-analysis	43 observational studies; n = 35,801	Folate status and dietary intake	Individuals with depression had lower folate levels (Hedges’ g, −0.24; 95% CI, −0.31 to −0.16).		[130]
	Meta-analysis	20 RCTs; n = 2256; adults with MDD or mild to severe depressive symptoms	Supplementation of folic acid or L-methylfolate	Supplementation with folic acid or L-methylfolate decreased depression scores.		[131]
	Meta-analysis	6 RCTs; patients with depression	Adjunctive folic acid or L-methylfolate	Adjunctive supplementation reduced HDRS scores (MD, −2.16; 95% CI, −3.62 to −0.69) and combined HDRS/BDI-II scores (SMD, −0.61; 95% CI, −0.97 to −0.24).		[132]
	Meta-analysis	11 RCTs; adults	Supplementation of folate and vitamin B12	Prolonged consumption, but not short-term use, of folate and vitamin B12 decreased the onset of clinical symptoms in people at depression risk (RR, 0.65; 95% CI, 0.43 to 0.98) or the relapse risk in adults with MDD (OR, 0.33; 95% CI, 0.12 to 0.94).		[133]
	Meta-analysis	16 RCTs; n = 6276 participants	Supplementation of folate and vitamin B12 with or without B6	Supplementation of folate and vitamin B12 with or without vitamin B6 had no effect on depression.		[134]
Vitamin C	Meta-analysis	25 observational studies; n = 91,666	Dietary vitamin C	Dietary vitamin C intake was lower in participants with depression compared to controls (WMD, −11.58; 95% CI, −14.8 to −8.29).	Consistent observational associations; limited and inconsistent intervention evidence	[139]
	Meta-analysis	Observational studies	Dietary and blood vitamin C	Patients with depression had a lower vitamin C intake (SMD, −0.18; 95% CI, −0.24 to −0.13) and a lower blood vitamin C level (SMD, −0.52; 95% CI, −0.94 to −0.10) compared to controls.		[140]
	Meta-analysis	10 RCTs; n = 836	Vitamin C supplementation	Overall effects were not significant, but a subgroup analysis showed modest improvement in participants with subclinical depressive symptoms who were not receiving antidepressants (Hedges’ g, −0.18; 95% CI, −0.35 to −0.01).		[141]
Magnesium	Dose–response meta-analysis	13 studies (10 cross-sectional and 3 cohort); n = 63,214; Adults	Dietary Mg intake	Higher dietary Mg intake was associated with a lower risk of depression (highest vs. lowest: RR, 0.66; 95% CI: 0.57 to 0.78); each 100-mg/day increase was associated with a 7% lower risk (RR, 0.93; 95% CI: 0.90 to 0.96).	Observational associations reported; limited and conflicting intervention evidence.	[145]
	SR and meta-analysis	58 studies; n = 45,062; mixed population	Dietary Mg, Mg status, Mg supplementation	Higher dietary Mg intake was associated with lower odds of depression in cross-sectional studies (OR, 0.66), but not in prospective studies.		[146]
	RCT meta-analysis	7 RCTs; n = 325; Adults with depressive disorder	Mg supplementation vs. placebo	Mg supplementation significantly reduced depression scores (SMD, −0.92; 95% CI: −1.44 to −0.40).		[147]
	RCT	Depressed patients with Mg deficiency	Mg oxide 500 mg/day vs. placebo, 8 wk	Mg supplementation produced a greater reduction in BDI-II scores than placebo (*p* = 0.02)		[171]
Zinc	Meta-analysis	9 zinc studies	Dietary zinc intake	Higher dietary zinc intake was associated with a lower risk of depression (highest vs. lowest: RR, 0.67; 95% CI: 0.58 to 0.76).	Observational associations reported; limited evidence for adjunctive benefit	[150]
	SR and meta-analysis	16 observational studies; MDD vs. controls	Serum zinc	Serum zinc levels were lower in individuals with MDD than in healthy controls (SMD, −0.62; 95% CI: −0.78 to −0.46)		[152]
	Clinical guideline	N/A—clinical guideline, Unipolar depression	Adjunctive zinc	Adjunctive zinc was provisionally recommended (++) for unipolar depression.		[154]
Iron	Meta-analysis	3 iron studies	Dietary iron intake	Higher dietary iron intake was associated with a lower risk of depression (highest vs. lowest: RR, 0.57; 95% CI: 0.34 to 0.95).	Limited observational evidence; insufficient evidence for antidepressant efficacy of supplementation.	[150]
	SR and meta-analysis	16 observational; MDD vs. controls	Serum iron	Serum iron levels were lower in individuals with MDD than in healthy controls (SMD, −0.36; 95% CI: −0.52 to −0.20).		[152]
	Umbrella review	34 meta-analyses; 46 health outcomes; mixed population	Total dietary iron intake	Higher total dietary iron intake was associated with a lower risk of depression (RR, 0.57; 95% CI, 0.34 to 0.95)		[172]
	RCT	n = 198, non-anemic women with low ferritin and fatigue	Iron 80 mg/day vs. placebo, 12 wk	Iron supplementation improved fatigue but did not improve depression (*p* = 0.97) or anxiety (*p* = 0.50).		[160]
Polyphenols	Meta-analysis	18 RCTs; n = 1523	Polyphenol supplementation	Polyphenol supplementation improved depression scores (SMD, −0.95; 95% CI, −1.76 to −0.13).	Limited and heterogeneous intervention evidence; clinical efficacy of individual polyphenols remains uncertain.	[165]
	Meta-analysis	10 studies	Flavonoids	Flavonoids attenuated depression symptoms (Hedges’ g, −0.49; 95% CI, −0.81 to −0.16).		[167]
	Meta-analysis	6 RCTs; n = 377; patients with depression	curcumin	Curcumin treatment reduced HDRS scores in patients with depression (SMD, −0.34; 95% CI, −0.56 to −0.13).		[168]
	Systematic review	22 animal studies	resveratrol	Resveratrol ameliorated depressive symptoms in rodents.		[169]

^1^ Clinical interpretation represents qualitative narrative assessments based on the type, consistency, and limitations of the available evidence and are not formal certainty-of-evidence ratings. BDI-II, Beck Depression Inventory-II; CI, confidence interval; DHA, docosahexaenoic acid; EPA, eicosapentaenoic acid; HDRS, Hamilton Depression Rating Scale; HR, hazard ratio; MD, mean difference; MDD, major depressive disorder; OR, odds ratio; RCT, randomized controlled trial; RR, risk ratio; SMD, standardized mean difference; SNRI, serotonin–norepinephrine reuptake inhibitor; SSRI, selective serotonin reuptake inhibitor; WMD, weighted mean difference N/A, not applicable.

## 6. Conclusions and Future Perspectives

Nutritional psychiatry has emerged as an interdisciplinary field examining the relationship between nutrition and mental health. Depression is a heterogeneous disorder involving interacting neurobiological, neuroimmune, and metabolic processes, including alterations in neurotransmission, neuroinflammation, oxidative stress, mitochondrial function, gut–brain communication, HPA-axis regulation, and immunometabolic homeostasis. These processes provide biological plausibility for investigating the relationship between nutrition and depression, but mechanistic plausibility alone does not establish clinical efficacy.

As summarized in this review, observational evidence generally supports associations between overall dietary patterns and depression-related outcomes. Greater adherence to the Mediterranean diet is generally associated with more favorable depression-related outcomes, whereas Western dietary patterns and greater consumption of UPFs are associated with less favorable outcomes. Evidence concerning the DASH diet is more limited but also suggests potential associations with depressive symptoms. These findings highlight the relevance of overall dietary patterns, which reflect the combined contributions of multiple foods, nutrients, and bioactive compounds. Associations have also been reported for individual nutrients, including omega-3 fatty acids, vitamin D, vitamin C, folate, vitamin B12, vitamin B6, and essential minerals. However, the consistency and clinical relevance of these associations vary across nutrients, and the independent contributions of specific nutrients to depression-related outcomes remain uncertain.

Importantly, observational associations do not necessarily translate into clinical benefits from dietary modification or nutrient supplementation. Although some dietary interventions and nutrient supplementation trials have demonstrated potential benefits for depressive symptoms in selected populations, these findings have not been consistently replicated across trials. Large prevention trials involving vitamin D, omega-3 fatty acids, and multinutrient supplementation have reported null or unfavorable findings regarding depression prevention [87,99,118]. MR studies provide a complementary approach to investigating potential causal relationships, although the available findings for specific nutritional exposures remain limited and inconclusive [113]. These discrepancies highlight the importance of distinguishing mechanistic plausibility, observational associations, and clinical intervention evidence, as well as differentiating the treatment of depressive symptoms from the prevention of incident depression. Current evidence does not support nutritional interventions as established alternatives to psychiatric or psychotherapeutic treatments, although they may have a potential adjunctive role in selected populations.

The heterogeneity of depression may partly contribute to variability in nutritional intervention outcomes. Baseline nutritional status, depression phenotype, adiposity, metabolic health, and inflammatory profiles may influence responses to dietary or nutrient interventions. In this context, IMD provides a potential framework for investigating whether nutritional interventions have differential effects across biologically distinct subgroups. However, direct clinical evidence supporting phenotype-specific responses remains limited.

Future research should aim to identify which individuals are most likely to benefit from specific nutritional interventions and whether nutritional, biological, or clinical characteristics can reliably inform treatment selection. Future intervention studies should also distinguish correction of confirmed nutritional deficiencies from supplementation in nutritionally sufficient individuals. Integrating nutritional assessment with genetic, metabolic, inflammatory, and gut microbiome data using multi-omics approaches may enable more personalized nutritional strategies. AI and machine-learning approaches may assist in integrating these complex data; however, their clinical utility for predicting responses to nutritional interventions in depression remains to be established [173]. Ultimately, well-designed and adequately powered clinical trials incorporating baseline nutritional status and biological phenotyping are needed to determine the role of precision nutrition in the prevention and adjunctive management of depression.

## Data Availability

No new data were created or analyzed in this study. Data sharing is not applicable to this article.
